# DA-CapsNet: dual attention mechanism capsule network

**DOI:** 10.1038/s41598-020-68453-w

**Published:** 2020-07-09

**Authors:** Wenkai Huang, Fobao Zhou

**Affiliations:** 1 Center for Research On Leading Technology of Special Equipment, School of Mechanical and Electrical Engineering, Guangzhou University, Guangzhou, 510006 China; 2 School of Mechanical and Electrical Engineering, Guangzhou University, Guangzhou, 510006 China

**Keywords:** Machine learning, Image processing, Network models

## Abstract

A capsule network (CapsNet) is a recently proposed neural network model with a new structure. The purpose of CapsNet is to form activation capsules. In this paper, our team proposes a dual attention mechanism capsule network (DA-CapsNet). In DA-CapsNet, the first layer of the attention mechanism is added after the convolution layer and is referred to as Conv-Attention; the second layer is added after the PrimaryCaps and is referred to as Caps-Attention. The experimental results show that DA-CapsNet performs better than CapsNet. For MNIST, the trained DA-CapsNet is tested in the testset, the accuracy of the DA-CapsNet is 100% after 8 epochs, compared to 25 epochs for CapsNet. For SVHN, CIFAR10, FashionMNIST, smallNORB, and COIL-20, the highest accuracy of DA-CapsNet was 3.46%, 2.52%, 1.57%, 1.33% and 1.16% higher than that of CapsNet. And the results of image reconstruction in COIL-20 show that DA-CapsNet has a more competitive performance than CapsNet.

## Introduction

Convolutional neural networks (CNNs) are widely used in computer vision because of their great success in target recognition and classification. However, CNNs are not perfect, and their ability to deal with the spatial relationships of image entities is inadequate. Routing refers to the process that determines the network scope of the end-to-end path of the packet from the source to the destination. In neural networks, routing is the process of transferring information from one layer to another. In CNNs, after the convolution operation of each layer is completed, a pooling operation is carried out. A pooling operation can be regarded as a routing application. While local characteristics are improved, other internal information, such as position and attitude, can be lost. When the recognition changes the image of position feature, the result of CNNs is not good. For example, when processing an image with a nose, eyes, mouth, and other facial features but not a face, the CNN will stubbornly think that it is a face^1,2^. In order to solve the problem of the traditional CNN being too coarse for image understanding, Hinton et al. proposed a CNN with dynamic routing algorithm^3,4^.

Capsule networks (CapsNets) are effective at recognizing various attributes of specific entities in the image, including pose (position, size, direction), deformation, speed, reflectivity, hue, texture, and so on. When recognizing the image, CapsNets can judge that the image is not a face. The ability of a CapsNet to recognize image attributes depends on the characteristics of the capsules. The higher the level of a capsule, the more information it grasps. The dynamic routing algorithm is used to change low-level capsules into high-level capsules. High-level capsules contain a large amount of image information. It has been found that there is room to improve the level for original high-level capsules. In this paper, our team proposes a dual attention mechanism capsule network (DA-CapsNet). DA-CapsNet has two layers of attention modules; one layer acts as the convolution layer, and the other acts as the PrimaryCaps layer. The purpose of DA-CapsNet is to improve the important information in the capsules, reduce the non-important information, increase the contribution of important information to the capsules, and improve the hierarchy of the capsules. Compared with the original capsule, the improved capsule has more entity attributes and image information.

Our team studied the performance of DA-CapsNet for MNIST, CIFAR10, FashionMNIST, SVHN smallNORB and COIL-20 classification tasks. The results show that the performance of DA-CapsNet is better than that of CapsNet, and it has higher classification accuracy.

Overall, the main contributions of our work are twofold:

- The structure of the CapsNet is improved, and DA-CapsNet based on a double attention mechanism is proposed.
- DA-CapsNet can generate capsules with a higher level, thus effectively improving the accuracy of classification.

## Related work

### CapsNet

The traditional deep neural network cannot effectively obtain the structure of image entity attributes^5–7^, which leads to inadequate results when performing some tasks. In order to make the neural network recognize the spatial information of the image space, Hinton et al.^4^ proposed the concept of a capsule. Following that, Sabour et al.^3^ realized CapsNet for the first time, and many new researches have promoted its development. Shahroudnejad et al.^8^ further explained CapsNet. Jaiswal et al.^9^ proposed CapsuleGAN, which uses CapsNet instead of a CNN as the discriminator and is a combination of CapsNet and the popular antagonistic neural network. In terms of computer vision, CapsNet has been used to detect fake images and videos^10^, recognize human movements, learn time information from spatial information^11^, encode facial actions^12^, and classify hyperspectral images^13^, all of which are based on its recognition of image entity attributes. In natural language processing, Zhang et al.^14^ using capsule network to extract relationship, Du et al.^15^ proposed a new hybrid neural network based on emotion classification capsules, and McIntosh et al.^16^ applied multimodal capsule routing to action video segmentation. In medicine, CapsNet has been used to predict Alzheimer disease^17^, automatically classify apoptosis^18^, identify sign language^19^, and classify brain tumor types^20^.

### Attention mechanism

A visual attention mechanism is a special brain signal processing mechanism in human vision^21^. When human vision captures an image, it automatically focuses on an interesting part, invests more energy in and obtains more information from the relevant areas, and suppresses other irrelevant information. Researchers have incorporated the concept of visual attention into the field of computer vision to improve the efficiency of the model^22–25^. In essence, the neural network is a function approver^26^. The structure of the neural network determines what kind of function it can fit^27,28^. Generally, A typical neural network can be implemented as a series of matrix multiplexes and element-wise non-linearities, where the elements of the input or eigenvectors interact only by addition^29–31^. In theory, neural networks can fit any function, but in reality, the fitted functions are limited. Zhang et al.^32^ proposed that spatial interaction in human visual cortex requires multiplication mechanism. Swindale et al.^33,34^ proposes that the forward information in cortical maps is directed to the backward information by a attentional mechanism that allows control of the presence of multiplication effects and multiplicative interactions. By introducing an attention mechanism, the functions of input vectors are computed with the mask used to multiply the feature to reduce the limitations of neural network fitting functions, which extends the operation of input vectors to multiplication.

## Methods

### Background

**Figure 1:**
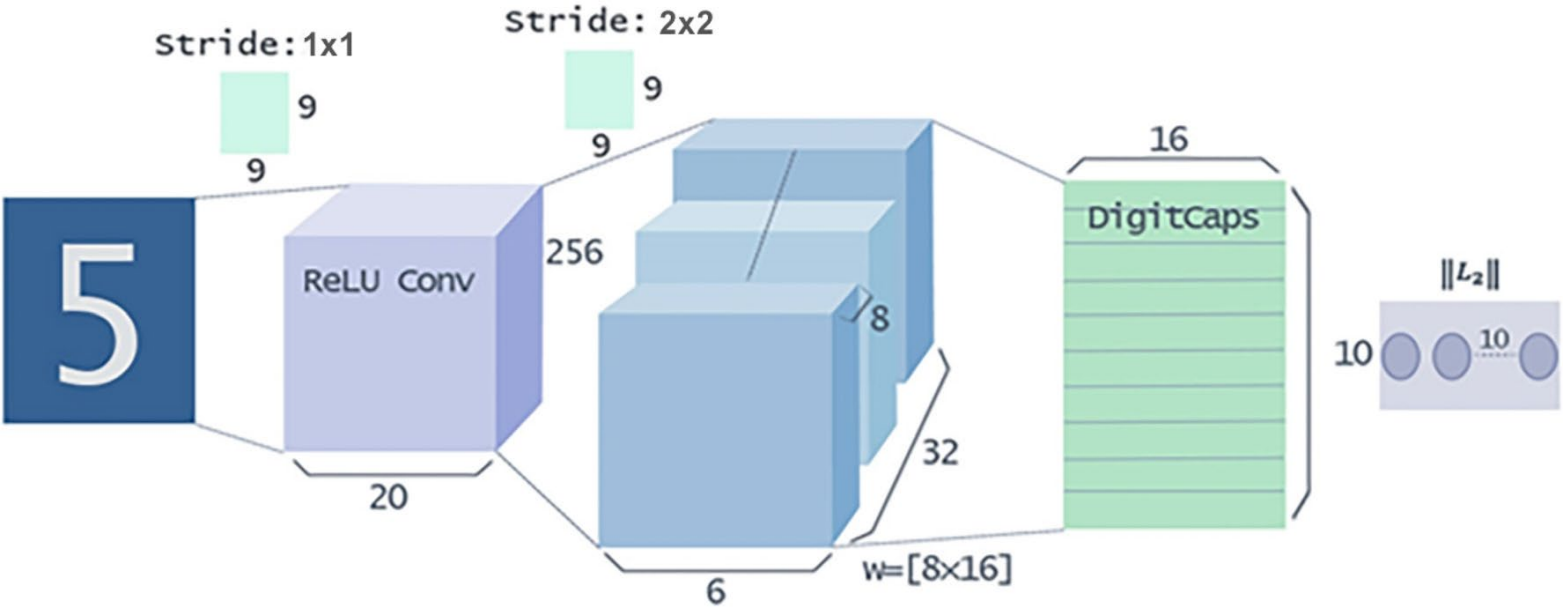
CapsNet architecture.

In deep learning, capsules are sets of embedded neurons, and a CapsNet is comprised of these capsules. The activity vector of a capsule represents the instantiation parameter of a specific type of entity, such as a target or part of a target. Figure 1 is the original CapsNet structure diagram, which shows the comparable results of a deep convolution network. The length of the activation vector of each capsule in the DigitCaps layer represents the presentation of each class instance and is used to calculate the classification loss.

**Figure 2:**
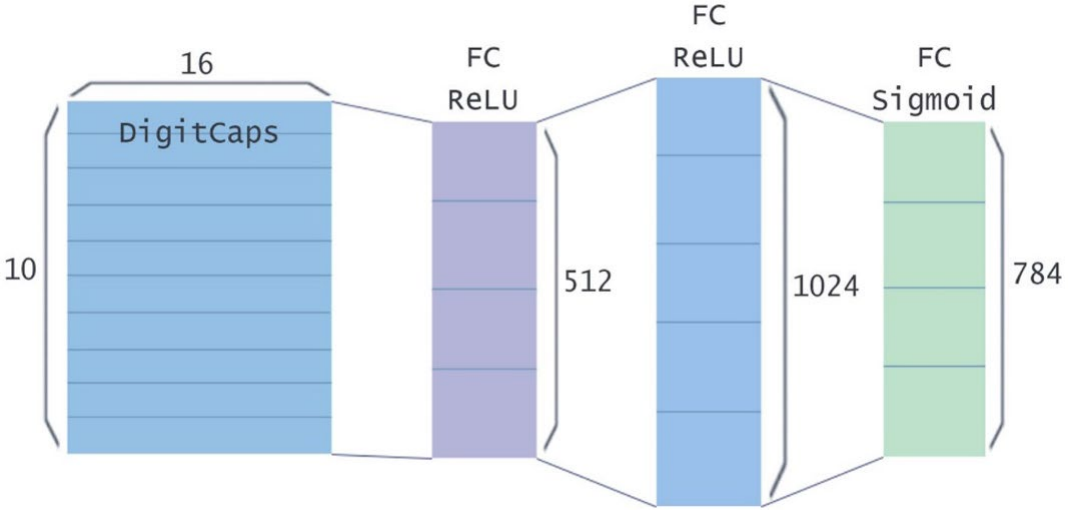
Reconstructing a decoding structure from the DigitCaps layer.

Figure 2 shows the decoding structure of the DigitCaps layer. DigitCaps pass through two full connection layers controlled by ReLU and tanh. The euclidean distance between images and the output of the sigmoid layer are minimized in training. Using the correct label as the reconstruction target in the training.

The length of the capsule output vector represents the probability that the entity represented by the capsule exists in the current input. Therefore, a nonlinear squashing function is used as the activation function to ensure that the short vector is compressed to a length close to 0 and the long vector is compressed to a length slightly less than 1. In the original paper^3^, the constant used in the squashing function was 1. In the experiment, the constant was changed to 0.5 to improve the scaling scale of the squashing function. Table 1 shows the accuracy of CapsNet in each dataset under different squashing constant. A squashing function was calculated using Eq. (1) and the scaling function were calculated using Eqs. (2) and (3):

1 $$\begin{array}{c}{V}_{j}=Squash\left({S}_{j}\right)=\frac{{\Vert {S}_{j}\Vert }^{2}}{1+{\Vert {S}_{j}\Vert }^{2}}\frac{{S}_{j}}{\Vert {S}_{j}\Vert }\to \frac{{\Vert {S}_{j}\Vert }^{2}}{0.5+{\Vert {S}_{j}\Vert }^{2}}\frac{{S}_{j}}{\Vert {S}_{j}\Vert }\end{array}$$

2 $$\begin{array}{c}{z}_{1}=\frac{{\Vert {S}_{j}\Vert }^{2}}{1+{\Vert {S}_{j}\Vert }^{2}}\end{array}$$

3 $$\begin{array}{c}{z}_{2}=\frac{{\Vert {S}_{j}\Vert }^{2}}{0.5+{\Vert {S}_{j}\Vert }^{2}}\end{array}$$

where ${V}_{j}$ is the output vector of the $jth$ capsule, ${S}_{j}$ is the input vector of the $jth$ capsule, and $\Vert {S}_{j}\Vert$ is the module length of the vector ${S}_{j}$.

**Table 1:** Accuracy of datasets with different squashing constant.

Squashing constant	CIFAR10 (%)	FashionMNIST (%)	SVHN (%)
1.0	82.45	90.82	90.78
0.5	82.95	92.41	91.36
Improvement	**0.50**	**1.59**	**0.58**

**Figure 3:**
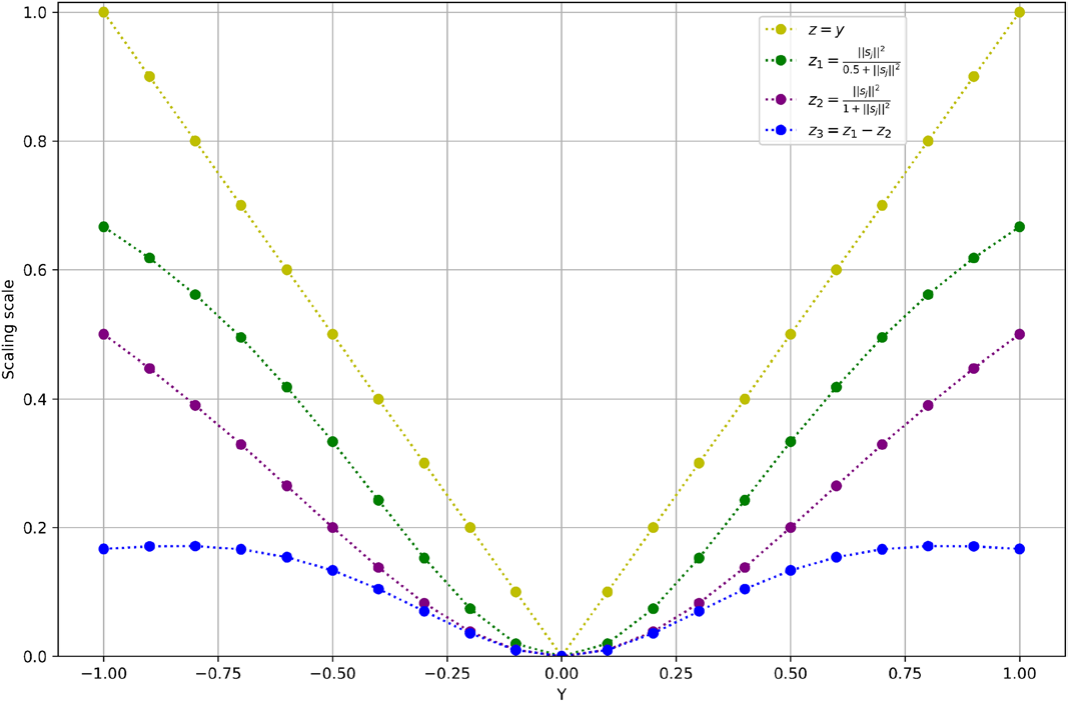
Comparison of two different squashing functions.

It can be seen from Fig. 3. That when the norm of vector is small and large, the scaling scale of ${z}_{1}$ function increases less, and when the norm of vector is in the middle, the scaling scale increases more. After the activation function is changed, the network’s attention to image features is increased to achieve better results.

The input of CapsNet ${S}_{j}$ is calculated with Eq. (4):

4 $$\begin{array}{*{20}c} { S_{j} = \mathop \sum \limits_{i} c_{ij} \hat{u}_{j|i} } \\ \end{array} ,$$

and ${\widehat{u}}_{j|i}$ is calculated with Eq. (5):

5 $$\begin{array}{*{20}c} {\hat{u}_{j|i} = W_{ij} u_{i} } \\ \end{array} .$$

The total input of a capsule ${S}_{j}$ is a weighted sum of all prediction vectors ${\widehat{u}}_{j|i}$ from the capsule of the lower layer, and ${u}_{i}$ is the output of a capsule of the lower layer multiplied by a weight matrix ${W}_{ij}$. The coupling coefficient ${c}_{ij}$ is determined by the iterative dynamic routing process, calculated using Eq. (6).

6 $$\begin{array}{*{20}c} {c_{ij} = \frac{{\exp \left( {b_{ij} } \right)}}{{\mathop \sum \nolimits_{k} \exp \left( {b_{ik} } \right)}}} \\ \end{array} .$$

To get ${c}_{ij}$, ${b}_{ij}$ must first be found; ${b}_{ij}$ is calculated using Eq. (7):

7 $$\begin{array}{*{20}c} {b_{ij} \leftarrow b_{ij} + \hat{u}_{j|i} \cdot v_{j} } \\ \end{array} .$$

The initial value of ${b}_{ij}$ is 0; from this we can get ${c}_{ij}$ and ${u}_{i}$, which is the output of the previous layer of capsules. With these three values, we can determine the next level of ${S}_{j}$. Hinton et al.’s^4^ experiments with MNIST showed that CapsNet has a unique effect in processing an image target or part of a target, which cannot be solved by traditional CNNs.

### DA-CapsNet

#### Overall structure of DA-CapsNet

**Figure 4:**
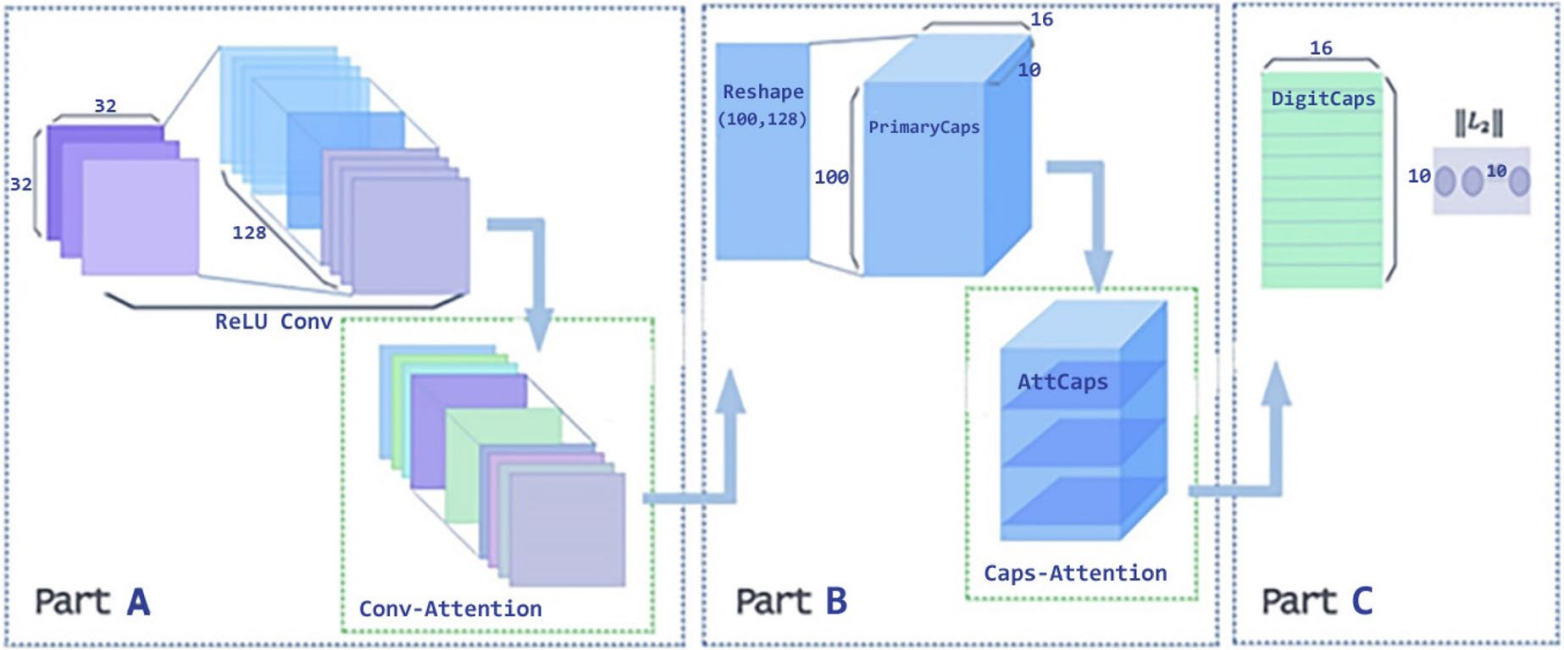
DA-CapsNet architecture. The network structure diagram (CIFAR10) shows two layers of attention mechanisms added to the official network model for keras^35^.

Figure 4 presents the architecture of DA-CapsNet. Unlike CapsNet, DA-CapsNet adds two layers of attention mechanisms, including Conv-Attention in ReLU-Conv to PrimaryCaps and Caps-Attention in PrimaryCaps to DigitCaps.

In Fig. 4, the purpose of Part A is to turn an image into a higher contribution attention convoy. The image is input with the dimensions of [32,32,3]. Using two layers of 3 × 3 step size 1 convolution kernel instead of 5 × 5 convolution check image to convolute to obtain more details of the image information, using ReLU to activate function, the result of convolution is a tensor of 10 × 10 with 128 channels ([10,10,128]). Then, through Conv-Attention processing, the image provides a higher contribution to the experimental results of attention convolution.

The purpose of Part B is to transform the PrimaryCaps into the more productive attcaps and then generate a DigitCaps that is higher level than in the original network. In this process, one-dimensional convolution is used instead of full connection operation to improve the operation efficiency. The activation function is linear, which generates 100[10,16] dimensional PrimaryCaps. Each capsule shares the weight. The PrimaryCaps is then processed by Caps-Attention to become attention capsules(AttCaps).

In Part C, the dynamic routing algorithm is used to change the AttCaps into DigitCaps. The length of the activation vector of each capsule in the DigitCaps layer represents the corresponding predicted probability of each class.

#### Attention mechanism in DA-CapsNet

The function of Conv-Attention is to transform the result of the first convolution into the attention convolution. The role of Caps-Attention is to change the PrimaryCaps into AttCaps. The purpose of the two attention modules is to make the capsule focus on a wider area of the image, get more information and improve the accuracy of classification. We will use the results of model reconstruction to prove that the dual attention mechanism makes the capsule pay more attention to the image information.

#### Conv-attention module

**Figure 5:**
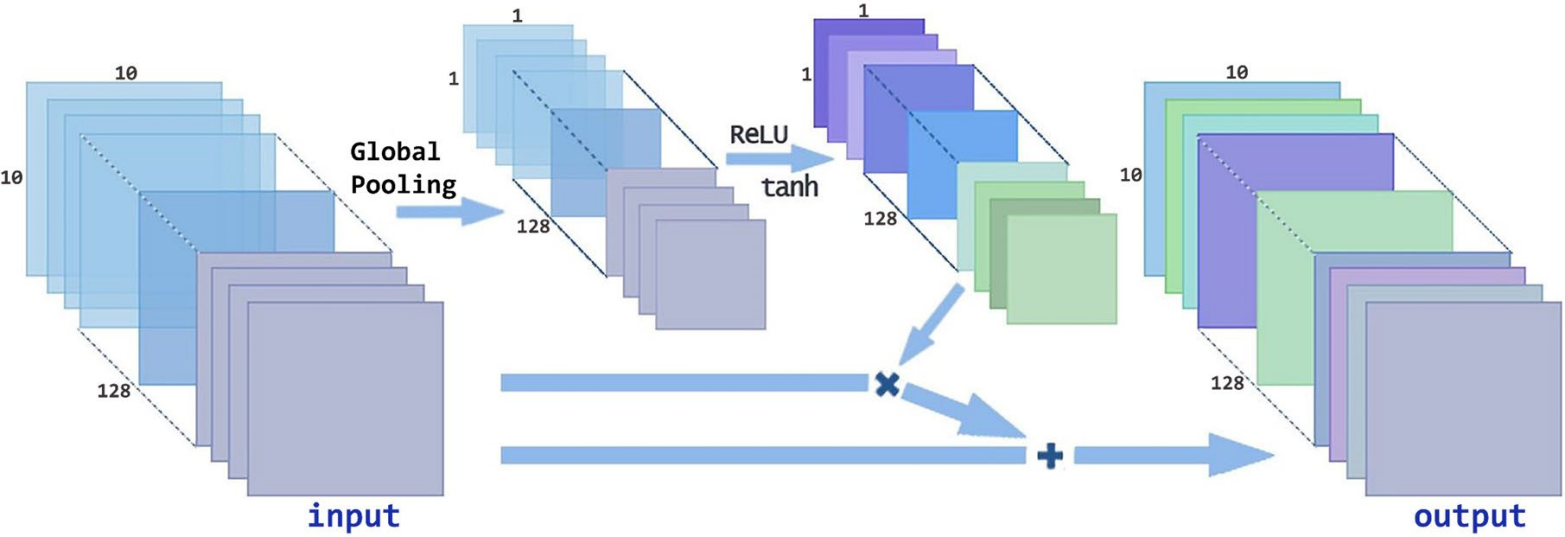
Conv-Attention architecture.

Figure 5 shows the principle of Conv-Attention. After the image is processed by ReLU Conv, the global pooling operation is carried out, which gathers the plane information into the point information, and then passes through the full connection neural network controlled by the relu and tanh activation functions, respectively. Finally, the convolution of attention is obtained by multiplying and adding the results of ReLU Conv. For the input setting $${u}_{pc}$$, $${the length of u}_{pc}$$ is M, the width is N, and the number of channels is Q. Thus, the dimensions is [M,N,Q]. The first step is to perform global pooling for each channel. The global pooling formula is calculated with Eq. (8):

8 $$\begin{array}{*{20}c} {u_{{gq_{k} }} = \frac{1}{MN}\mathop \sum \limits_{i = 0}^{M} \mathop \sum \limits_{j = 0}^{N} q_{kij} , for \quad q_{k} \in q} \\ \end{array} .$$

The resulting setting after global pooling is ${u}_{g}$. The shape of ${u}_{g}$ is [1,1,Q]. The second step is to synthesize and process the features extracted in the first step to improve the nonlinear expression ability of the model^36^. Here, two layers of full connection are used to process ${u}_{g}$ to get ${u}_{1}$ and ${u}_{2}$.The first activation function is ReLU, and the second activation function is tanh. These are calculated using Eqs. (9) and (10):

9 $$\begin{array}{*{20}c} {u_{1} = ReLU\left( {W_{1} u_{g} + b_{1} } \right)} \\ \end{array} ,$$

10 $$\begin{array}{*{20}c} {u_{2} = \tanh \left( {W_{2} u_{1} + b_{2} } \right)} \\ \end{array} ,$$

where ${u}_{1}$ and ${u}_{2}$ are the result of two layers of full connection, respectively.${W}_{1} and {W}_{2}$ are the corresponding weight matrix, and ${b}_{1} and {b}_{2}$ are the corresponding offset. After two full connection operations, the shape of ${u}_{2}$ is [1,1,Q].

In the third step, after ${u}_{2}$ is obtained, multiplying ${u}_{2}$ and ${u}_{pc}$ to get ${u}_{3}$, and then add ${u}_{3}$ and ${u}_{2}$ to get attention convolution ${u}_{c-att}$; ${u}_{c-att}$ and ${u}_{3}$ are calculated using Eqs. (11) and (12):

11 $$\begin{array}{*{20}c} {u_{c - att} = u_{pc} + u_{3} } \\ \end{array} ,$$

12 $$\begin{array}{*{20}c} {u_{3} = u_{pc} *u_{2} } \\ \end{array} .$$

#### Caps-attention module

**Figure 6:**
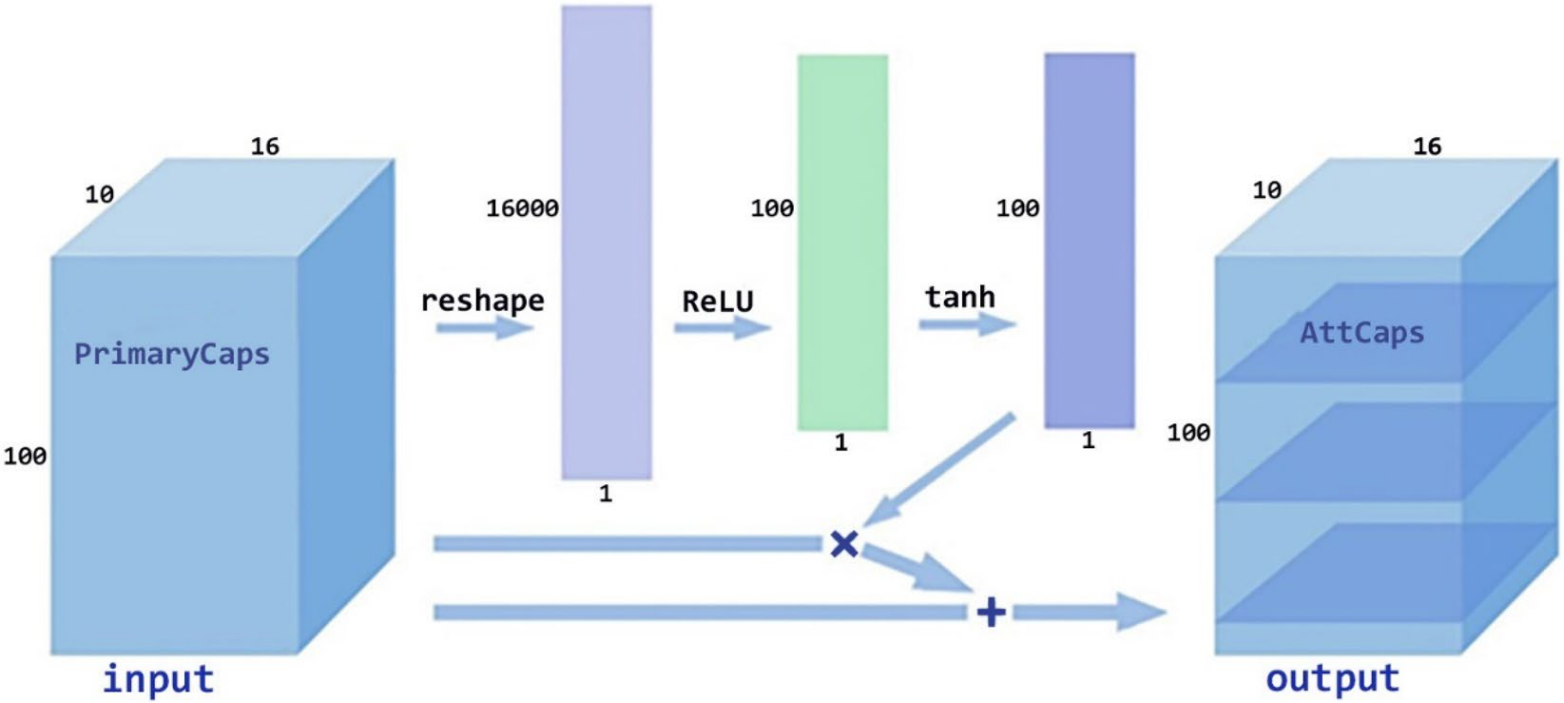
Caps-Attention architecture.

Figure 6 shows the principle of Caps-Attention. AttCaps is found by changing the shape of the PrimaryCaps, turning the PrimaryCaps into a vector, passing through the fully connected neural network controlled by the ReLU and tanh activation functions, and then multiplying and adding with the PrimaryCaps.

In the second step, ${u}_{pr}$ is fully connected twice to get ${u}_{p1}$ and ${u}_{p2}$ ^36^. The activation function of the first operation is ReLU, and the activation function of the second operation is tanh. Thus, ${u}_{pr}$ is calculated using Eqs. (13) and (14):

13 $$\begin{array}{*{20}c} {u_{p1} = ReLU\left( {W_{3} u_{pr} + b_{3} } \right)} \\ \end{array} ,$$

14 $$\begin{array}{*{20}c} {u_{p2} = \tanh \left( {W_{4} u_{p1} + b_{4} } \right)} \\ \end{array} ,$$

where ${u}_{p1}$ and ${u}_{p2}$ are the result of two layers of full connection, respectively.${W}_{3} and{ W}_{4}$ are the corresponding weight matrix, and ${b}_{3} and {b}_{4}$ are the corresponding offset.

In the third step, after ${u}_{p2}$ is obtained, multiplying ${u}_{p2}$ and ${u}_{p}$ to get ${u}_{p3}$, and then add ${u}_{p3}$ and ${u}_{p}$ to get attention capsules ${u}_{p-att}$; ${u}_{p-att}$ and ${u}_{p3}$ are calculated using Eqs. (15) and (16):

15 $$\begin{array}{*{20}c} {u_{p - att} = u_{p} + u_{p3} } \\ \end{array} ,$$

16 $$\begin{array}{*{20}c} {u_{p3} = u_{p} *u_{p2} } \\ \end{array} .$$

## Results

### Datasets and equipment

**Figure 7:**
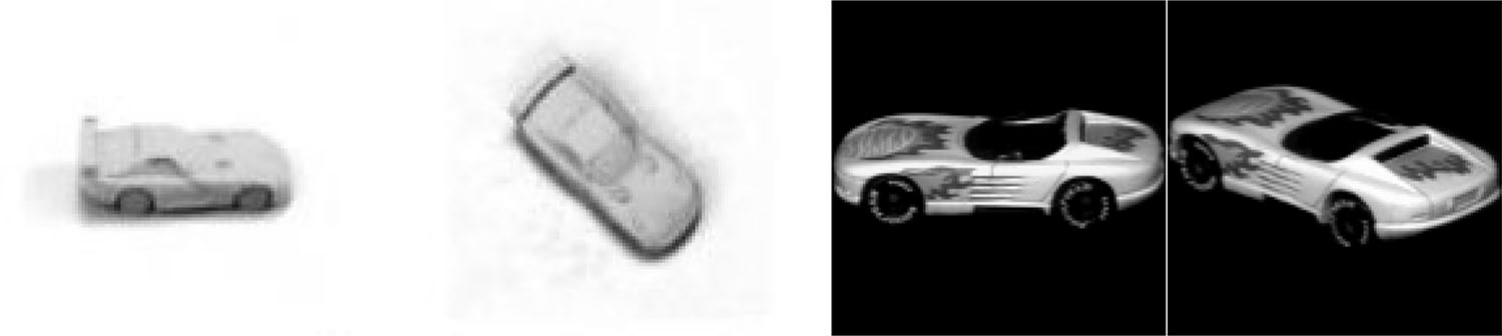
The left two images are smallNORB and the right two images are COIL-20.

In this experiment, the datasets used included MNIST, CIFAR10, FashionMNIST, SVHN, smallNORB, and COIL-20. MNIST is the most commonly used dataset for deep learning and includes 10 kinds of grayscale handwritten digital images. The CIFAR10 dataset consists of 60,000 32 × 32 color images of 10 classes, and each class has 6,000 images, 50,000 training images, and 10,000 test images. Based on the handwritten dataset MNIST, FashionMNIST includes 10 kinds of grayscale clothing images. SVHN is a collection of house numbers extracted from the Google Street View project. The SVHN dataset is much more complex. Some images may contain additional confusing numbers around the central numbers of interest. SmallNORB contains 50 images of toys, each of which is photographed in 18 different directions (0–340), 9 elevation angles and 6 lighting conditions, so each training and test set contains 24,300 images. COIL-20 is a collection of gray-scale images, including 20 objects from different angles, one image is taken every 5 degrees, each object has 72 images, and the data set contains a total of 1,440 images. The images of smallNORB and COIL-20 are shown in Fig. 7. We build our model use python3.6 and keras2.2.4, and spend 4 weeks training model with the GPU of NVIDIA Gtx1080ti and Windows operating system.

### Implementation details

The experiment was divided into four situations: no attention mechanism, Conv-Attention single-layer attention mechanism, Caps-Attention single-layer attention mechanism, and two-layer attention mechanism. All four cases were tested on each dataset. In these six datasets, preprocessing and real-time data expansion were carried out, and the number of image samples was increased through image transformation such as translation and flipping. For many attention mechanisms applied to image classification tasks, the last activation function mostly uses softmax or sigmoid, while the tanh function is used in experiments. The value range of the tanh function was (− 1,1).

### Image reconstruction

**Figure 8:**
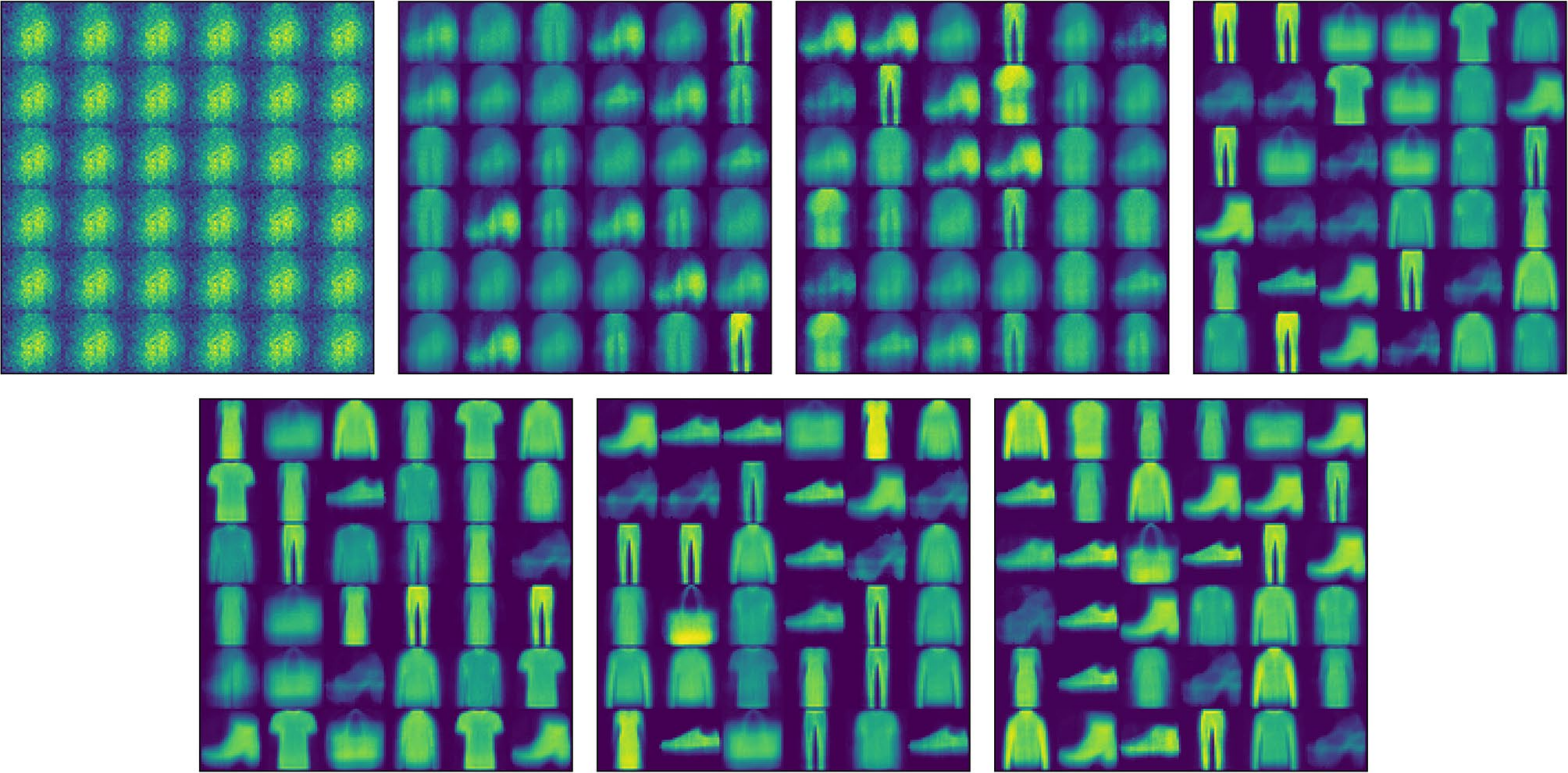
Images reconstructed by FashionMNIST on CapsNet.

**Figure 9:**
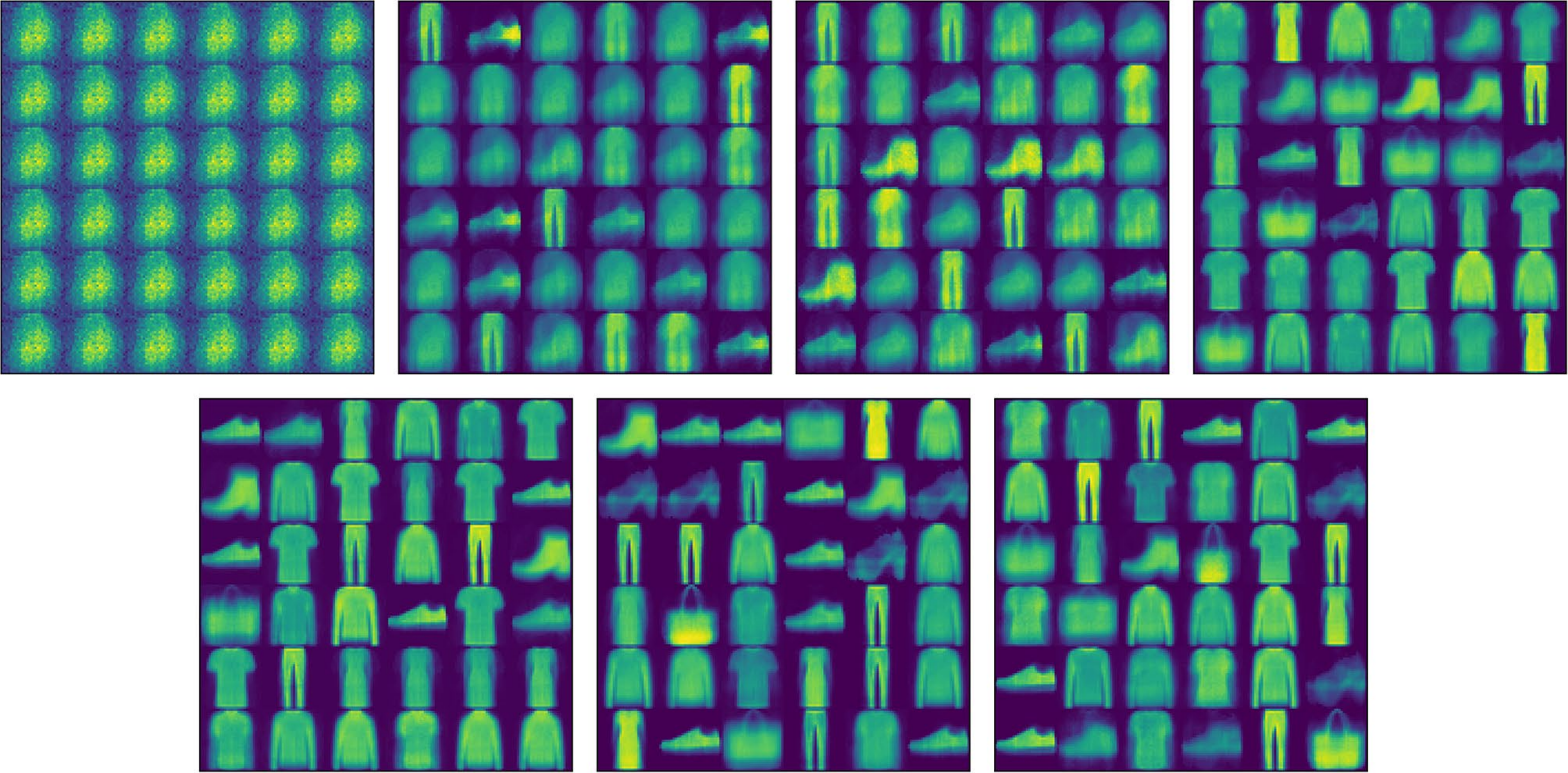
Images reconstructed by FashionMNIST on DA-CapsNet.

Figures 8 and 9 are the images reconstructed by FashionMNIST in CapsNet and DA-CapsNet respectively. We conducted image reconstruction experiments on FashionMNIST, trained 7 epochs, and took out the results of each epoch. Different network structures and weight changes during training lead to different results of the same dataset. From the visualization of the image, we can know that the working principle of the capsule is to analyze the whole image at first, and then focus on the features of the image gradually. Compared with Figs. 8 and 9, DA-CapsNet has more types of reconstruction and more obvious image features, and it can be seen that the image generated by DA-CapsNet has higher definition and less image noise. Under the action of Conv-Attention and Caps-Attention, the capsule not only speeds up the speed of focusing image features, but also pays attention to more information of the image, which makes the visualization of the reconstruction process clearer.

### MNIST results

**Figure 10:**
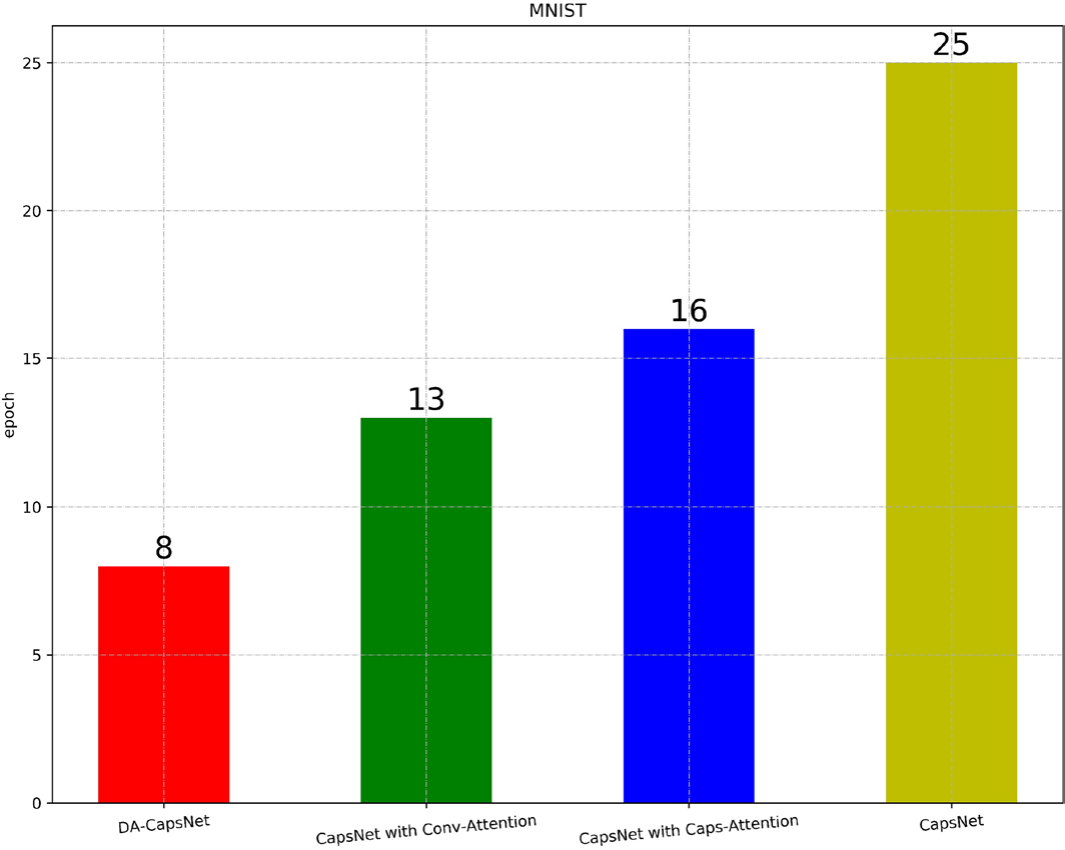
Classification accuracy for MNIST test dataset according to the epochs.

Figure 10 shows the epochs needed for four experiments to achieve 100% accuracy on MNIST test dataset. For MNIST, the experiment was based on the network structure of^4^ Fig. 4. The DA-CapsNet was trained on the training dataset and then input into the test dataset, and 100 epochs were run. As shown in Figs. 8,10 epochs were needed for DA-CapsNet to reach an accuracy of 100%, whereas 25 epochs were needed for CapsNet, 16 for CapsNet with Conv-Attention, and 13 for CapsNet with Caps-Attention.

### CIFAR10, SVHN and FashionMNIST results

**Figure 11:**
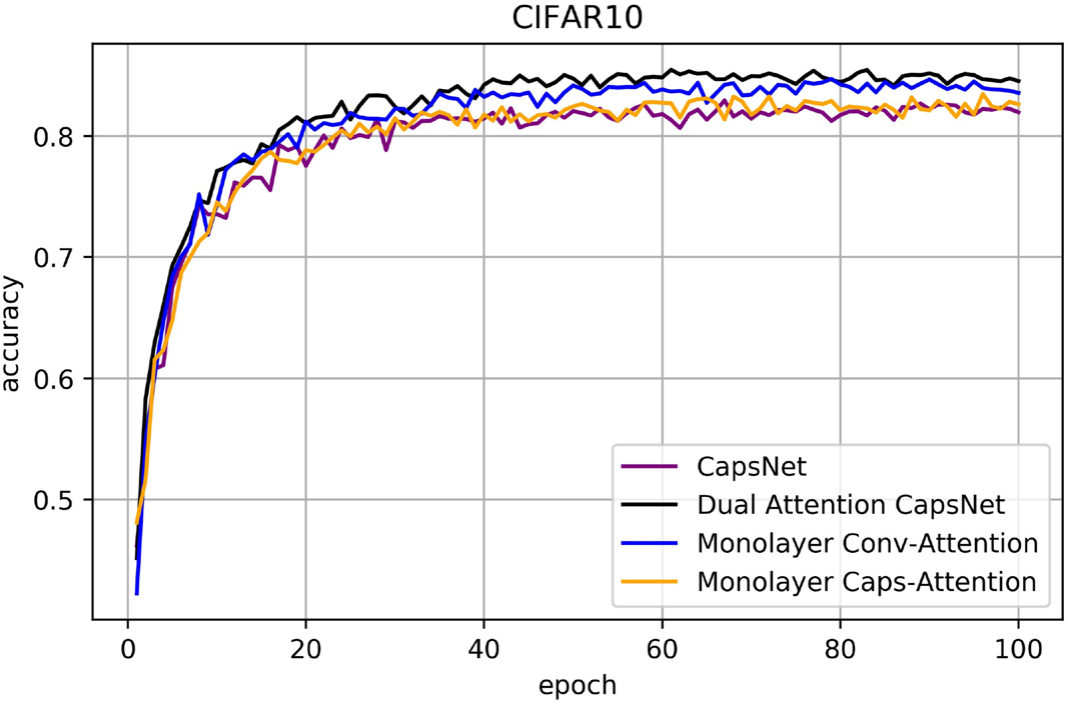
Classification accuracy for CIFAR10 test dataset according to the epochs.

**Figure 12:**
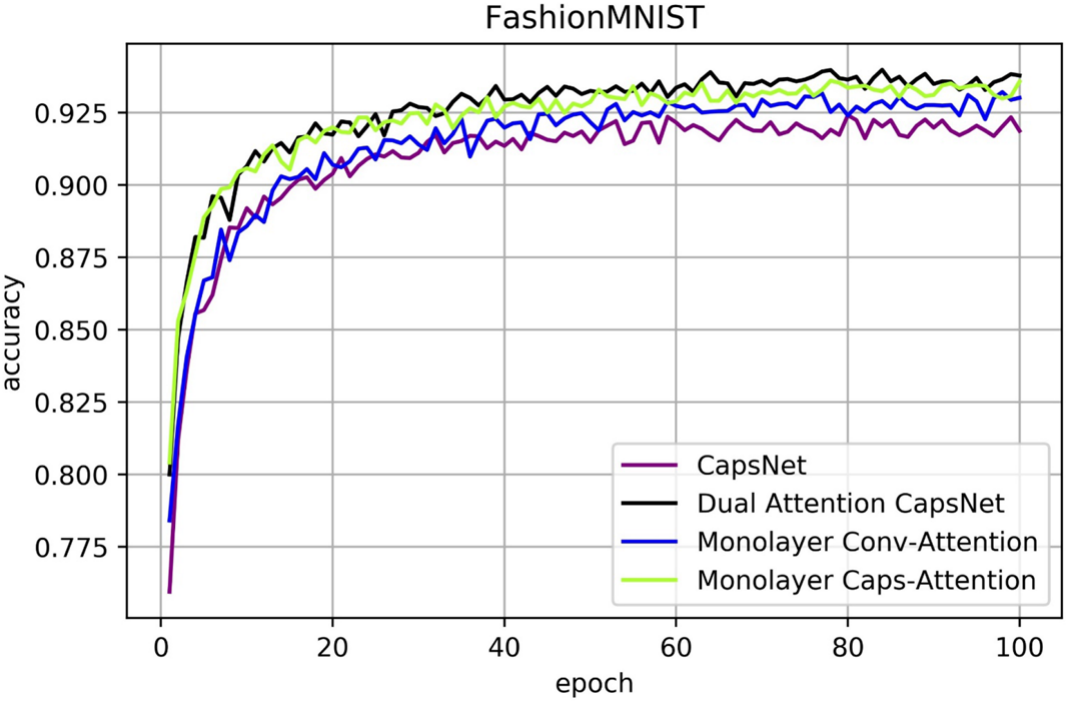
Classification accuracy for FashionMNIST test dataset according to the epochs.

**Figure 13:**
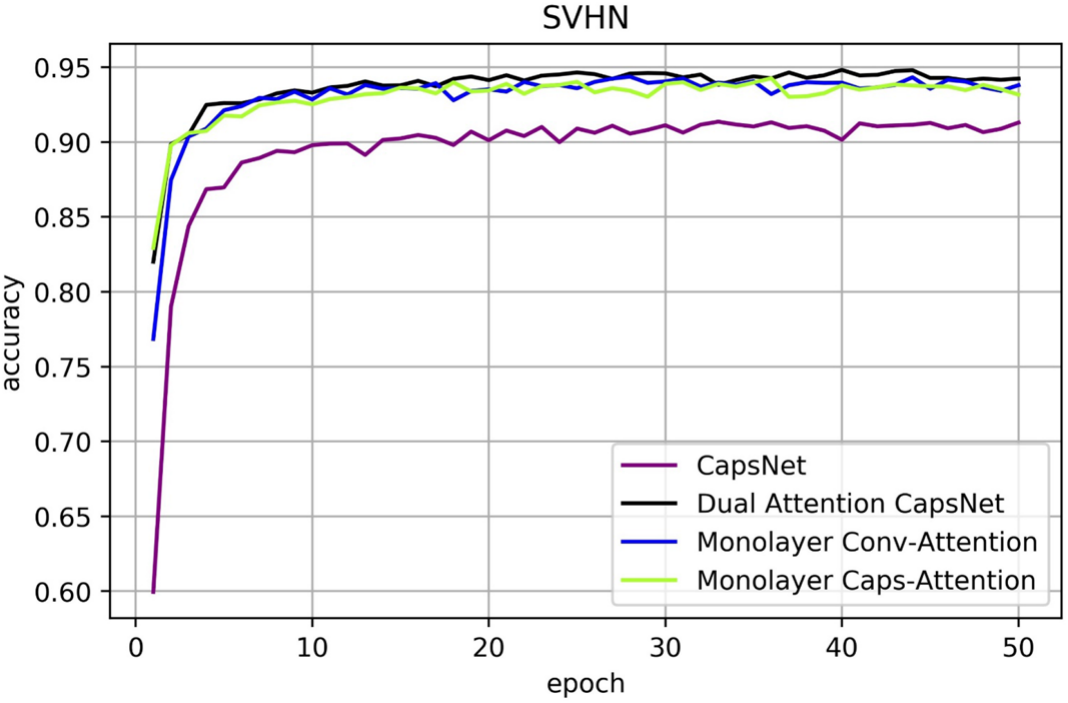
Classification accuracy on SVHN test dataset according to the epochs.

Figures 11, 12, 13 are line charts that demonstrate the accuracy of four experimental results using CIFAR10, SVHN and FashionMNIST, respectively. Table 2 shows the highest accuracy and improvement rate of four experiments in each dataset. Before processing the attention mechanism, the image is first convoluted. Table 3 shows the specific steps and methods of convolution. In FashionMNIST experiment, the input and output tensor was [10,10,128] through Conv-Attention and [10,100,16] through Caps-Attention. In CIFAR10 and SVHN experiment, the input and output tensor was [10,10,128] through Conv-Attention and [10,100,16] through Caps-Attention.

**Table 2:** Accuracy comparison for CIFAR10, SVHN and FashionMNIST for each network.

CIFAR10	Method	Conv-attention	Caps-attention	Accuracy (improvement)
	CapsNet			82.95%
	CapsNet	✓		84.71% (1.76%)
	CapsNet		✓	83.47% (0.52%)
	DA-CapsNet	✓	✓	85.47% (2.52%)
SVHN	CapsNet			91.36%
	CapsNet	✓		94.37% (3.01%)
	CapsNet		✓	94.26% (2.90%)
	DA-CapsNet	✓	✓	94.82% (3.46%)
FashionMNIST	CapsNet			92.41%
	CapsNet	✓		93.21% (0.80%)
	CapsNet		✓	93.60% (1.19%)
	DA-CapsNet	✓	✓	93.98% (1.57%)

**Table 3:** Formation of primarycaps for different datasets.

Image shape	[32, 32, 3] (SVHN, CIFAR10, smallNORB, COIL-20)	[28, 28, 1] (MNIST, FashionMNIST)
PrimaryCaps formation process	Conv2D( 64, (3, 3), activation = 'relu' ) Conv2D( 64, (3, 3), activation = 'relu' ) AveragePooling2D( (2, 2) ) Conv2D( 128, (3, 3), activation = 'relu' ) Conv2D( 128, (3, 3), activation = 'relu' ) Conv2D( 256, (3,3), activation = 'relu' ) Conv-Attention reshape	Conv2D( 64, (3, 3), activation = 'relu' ) Conv2D( 64, (3, 3), activation = 'relu' ) AveragePooling2D( (2, 2) ) Conv2D( 128,(2, 2), activation = 'relu ') Conv2D( 128, (2, 2), activation = 'relu' ) Conv-Attention reshape

### COIL-20 and smallNORB results

These two datasets are all sets of images taken by the same object from different angles. It is of great significance to study the unique spatial invariance of CapsNet. Compared with smallNORB, COIL-20 has more image categories, more features, such as texture, posture, and more differences between images. In the experiment, setting the image size of smallNORB and COIL-20 at 32 × 32 pixels. We choose the top ten categories of COIL-20 to experiment, and the ratio of training set to test set is 9:1, 100 epoch were run, and the batch_size is 16. At the same time, we trained a CNN as a baseline to compare with DA-CapsNet. CNN has two convolution layers including 32 and 64 channels respectively. Both layers have a kernel size of 5 and a stride of 1 with a 2 × 2 max pooling, and full connection layer with 1,024 unit with dropout. In smallNORB, CNN connects to 5-way softmax output layer, while in COIL-20, CNN is connected to 20-way softmax output layer.

**Figure 14:**
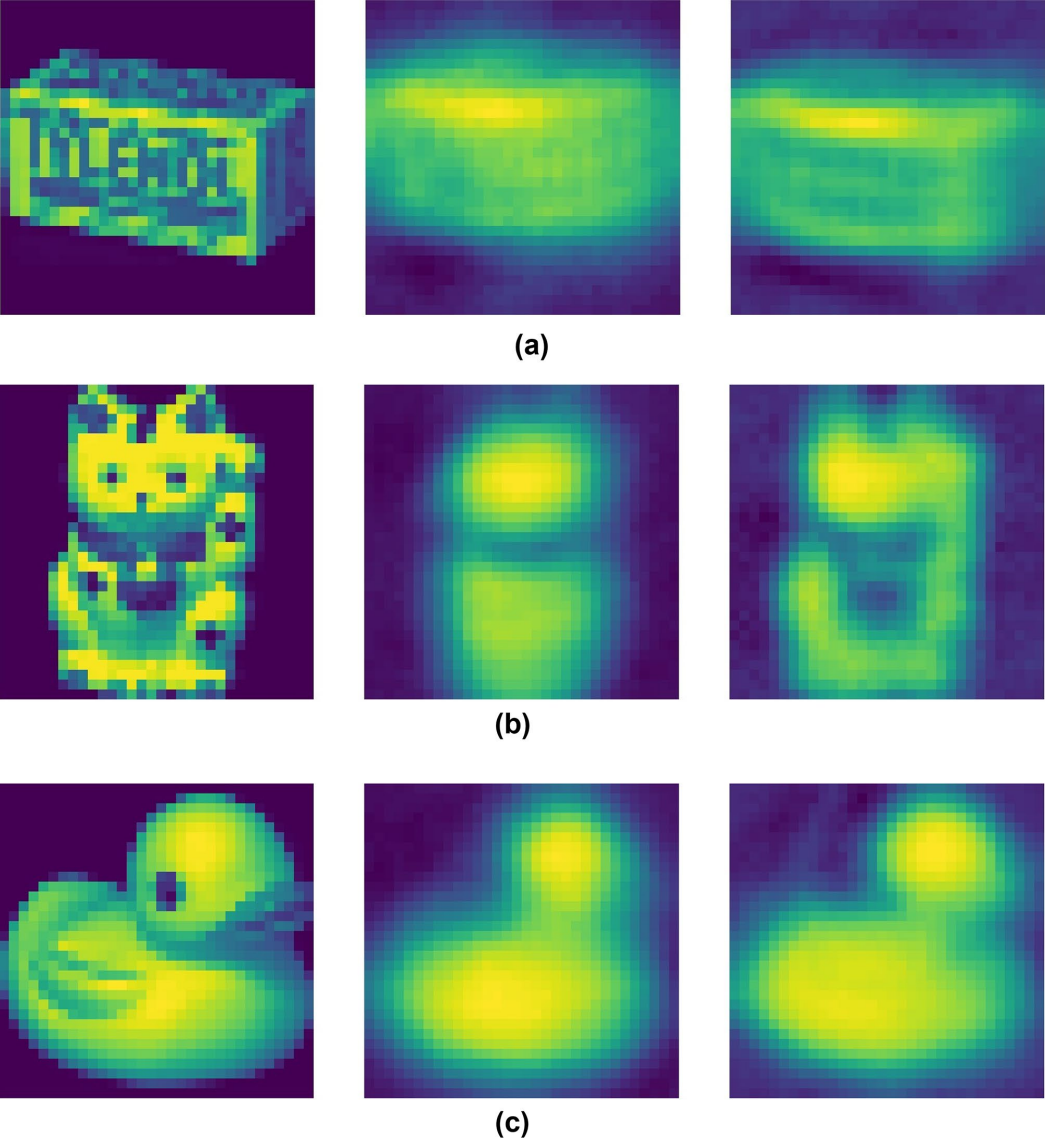
Reconstruction results of two neural network models. The left images is the image in COIL-20, the middle images is the result of CapsNet reconstruction, and the right images is the result of DA-CapsNet reconstruction.

Figure 14 shows the reconstruction results of DA-CapsNet and CapsNet in COIL-20. In Fig. 14a, the direction of image reconstructed by CapsNet tends to be horizontal, while DA-CapsNet is inclined, and the image reconstructed by DA-CapsNet contains more texture. The image of DA-CapsNet in Fig. 14b and c can more accurately express the posture and texture of plutus cat and duck. It can be seen that the design of DA-CapsNet has achieved the purpose of focusing more information on the image, and DA-CapsNet is more prominent in processing the characteristics of the image, such as direction, posture and texture. Table 4 shows the accuracy of CNN baseline on smallNORB and COIL-20, it also compares with CapsNet and DA-CapsNet. The accuracy of CNN baseline is 100% in COIL-20, 91.28% in smallNORB. In CapsNet, smallNORB and COIL-20 are 96.93% and 98.38 respectively, while DA-CapsNet achieves 98.26% and 100%.

**Table 4:** Comparison of the six datasets for CapsNet and DA-CapsNet.

Datasets	CNN baseline (%)	CapsNet (%)	DA-CapsNet (%)	Improvement (%)
MNIST	99.22	99.38	99.53	0.15
CIFAR10	72.20	82.95	85.47	2.52
SVHN	91.28	91.36	94.82	3.46
FashionMNIST	90.11	92.41	93.98	1.57
smallNORB	91.28	96.93	98.26	1.33
COIL-20	100	98.38	100	1.62

### All results

The results of CNN baseline, CapsNet and DA-CapsNet for the six datasets are summarized in Table 4. For the MNIST test dataset, the training results were all 100%, which is not easy to compare. Therefore, the average values of the first five epochs were used for the comparison. It can be seen from Table 4 that in MNIST, CIFAR10, SVHN, FashionMNIST, smallNORB, and COIL-20, DA-CapsNet showed an improved accuracy of 0.15%, 2.52%, 3.46%, 1.57%, 1.33%, and 1.16%, respectively, compared to CapsNet, and compared with CNN baseline, DA-CapsNet improves the accuracy significantly.

## Discussion

The effect of CapsNet depends on the characteristics of the capsule. The higher the level of the capsule, the more various attributes of the specific entity in the image, such as location, size, direction, etc. Improving the characteristics and enriching the content of capsules are the key goals of CapsNet research. On this basis, our study of DA-CapsNet focused on all the contents of the capsule, extracted the key content (enlarged the relevant parameters), discarded the non-key content (reduced the relevant parameters), improved the level of the capsule, and finally obtained the capsule with a larger proportion of key information.

In CapsNet, there is no uniform specification for the number of PrimaryCaps, which is determined artificially according to the convolution mode of the convolution layer, as can be seen from Table 3. The discreteness of the PrimaryCaps formed by the artificial convolution mode is strong, and the fitting function is subject to great limitation. The attention mechanism can be regarded as a multiplier to increase the number of functions that will fit the neural network model32-34. DA-CapsNet uses two levels of attention mechanism. In the network presented in this paper, the two attention mechanisms are in a series, and the results of the two attention mechanisms can be regarded as a composite function.

In Figs. 8, 9, 10, 11, 12, 13, More functions can be fitted by composite function than by multiplier, and the fitted function result is better than for a single attention level. At the same time, it can be seen that different attention layers have different effects depending on the network. For example, in the SVHN experiment, results were better for Conv-Attention than Caps-Attention, while in the FastionMNIST experiment, Caps-Attention had better results. The attention mechanism enables the neural network to focus on only a part of its input information, and it can select a specific input. The entities in the images had many attributes, such as posture, texture, etc. By adding two layers of attention mechanisms, the neural network can pay more attention to the information. The more CapsNet understands the entity characteristics of the image, the better its performance in the classification task.

## Conclusion

In this paper, our team proposed a CapsNet based on a double attention mechanism to improve the hierarchy of capsules, which was verified through six open datasets. The experimental results showed that DA-CapsNet with two attention mechanisms is better than CapsNet and a single attention mechanism for image classification. From the results of image reconstruction, DA- CapsNet pays more attention to image information faster and more accurately, have more outstanding ability to master image information. For SVHN, CIFAR10, FashionMNIST, smallNORB and COIL-20, the accuracy of DA-CapsNet was 3.46%, 2.52%, 1.57%, 1.33%, and 1.16% higher than that of CapsNet.

## References

[1] Deng F, Pu S, Chen X, Shi Y, Yuan T, Pu S (2018). Hyperspectral image classification with capsule network using limited training samples. Sensors.

[2] Wu,R.& Kamata, S.I. A jointly local structured sparse deep learning network for face recognition. *2016 IEEE International Conference on Image Processing (ICIP).* 3026–3030 (2016).

[3] Sabour, S., Frosst, N.& Hinton, G.E. Dynamic routing between capsules. In Proceedings of the 31st Conference on Neural Information Processing Systems(NIPS). 3859–3869 (2017).

[4] Hinton GE, Sabour S, Frosst N (2018). Matrix capsules with EM routing. Proc. Int. Conf. Learn. Represent..

[5] Oyallon, E. & Stephane, M. Deep roto-translation scattering for object classification. *Proc. IEEE Conf. Comput. Vision Pattern Recogn.* 2865–2873 (2015).

[6] Worrall, D.E., Garbin, S.J., Turmukhambetov, D.& Brostow, G.J. Harmonic networks: Deep translation and rotation equivariance. *Proc. IEEE Conf. Comput. Vision Pattern Recog*, 5028–5037 (2017).

[7] Cohen, T.& Welling, M. Group equivariant convolutional networks. in *Proc. IEEE Int. Conf. Mach. Learn*. 2990–2999 (2016).

[8] Shahroudnejad, A., Mohammadi, A.& Plataniotis, K.N. Improved explainability of capsule networks: Relevance path by agreement. *Proc. IEEE Global Conf. Signal Inf. Process. (GlobalSIP)*. 549–553 (2018).

[9] Jaiswal, A., AbdAlmageed, W., Natarajan, P. CapsuleGAN: Generative adversarial capsule network. Available at: https://arxiv.org/abs/1802.06167 (2018).

[10] Nguyen, H.H., Yamagishi, J. & Echizen, I. Capsule-forensics: Using capsule networks to detect forged images and videos. *Proc. IEEE Int. Conf. Acoust. Speech Signal Process. (ICASSP)*. 2301–2307 (2019).

[11] Algamdi, A.M., Sanchez, V. & Li, C.T. Learning temporal information from spatial information using CapsNets for human action recognition. in *IEEE Int. Conf. Acoust. Speech Signal Process*. 3867–3871 (2019).

[12] Ertugrul, I.O., Jeni, L.A.& Cohn, J.F. FACSCaps: Pose-Independent Facial Action Coding with Capsules. *2018 IEEE/CVF Conference on Computer Vision and Pattern Recognition Workshops (CVPRW)*. 2211–221109 (2018). 10.1109/CVPRW.2018.00287 PMC6443417 30944768

[13] Arun PV, Buddhiraju KM, Porwal A (2019). Capsulenet-based spatial-spectral classifier for hyperspectral images. IEEE J. Sel. Top Appl. Earth Observ Remote Sens..

[14] Zhang N, Deng S, Sun Z, Chen X, Zhang W, Chen H (2018). Attention-based capsule networks with dynamic routing for relation extraction. Proc. Conf. Empirical Methods Natural Lang. Process (EMNLP).

[15] Du YP, Zhao XZ, He M, Guo WY (2019). A novel capsule based hybrid neural network for sentiment classification. IEEE Access..

[16] McIntosh, B., Duarte, K., Rawat, Y.S., et al. Multi-modal capsule routing for actor and action video segmentation conditioned on natural language queries. Available at: https://arxiv.org/abs/1812.00303 (2018).

[17] Kruthika KR, Maheshappa HD (2019). Alzheimer’s Disease Neuroimaging Initiative. CBIR system using capsule networks and 3D CNN for Alzheimer’s disease diagnosis. Inform. Med. Unlocked..

[18] Mobiny A, Lu H, Nguyen HV, Roysam B, Varadarajan N (2019). Automated classification of apoptosis in phase contrast microscopy using capsule network. IEEE Trans. Med. Imag..

[19] Beşer, F., Kizrak, M.A., Bolat, B., et al. Recognition of sign language using capsule networks. In *2018 26th Signal Process. Commun. Appl. Conf. (SIU)*. 1–4 (2018).

[20] Afshar P, Mohammadi A, Plataniotis KN (2018). Brain tumor type classification via capsule networks. Proc. IEEE Int. Conf. Image Process. (ICIP).

[21] Yohanandan SA, Dyer AG, Tao D, Song A (2018). Saliencypreservation in low-resolution grayscale images. Eur. Conf. Comput. Vis. (ECCV)..

[22] Xu K, Ba J, Kiros R (2015). Show, attend and tell: Neural image caption generation with visual attention. Int. Conf. Mach. Learn..

[23] Jaderberg M, Simonyan K, Zisserman A, Kavukcuoglu K (2015). Spatial transformer networks. Proc. Int. Conf. Neural Inf. Process. Syst. (NIPS).

[24] Hu J, Shen L, Sun G (2018). Squeeze-and-excitation networks. Proc. IEEE Conf. Comput. Vis. Pattern Recognit..

[25] Xinyi, Z.& Chen, L. Capsule graph neural network. *ICLR*. (2019).

[26] Castro JL, Delgado M (1996). Fuzzy systems with defuzzification are universal approximators. IEEE Trans. Syst. Man Cybern..

[27] Wei Q, Jiang Y, Chen J (2018). Machine-learning solver for modified diffusion equations. Phys. Rev. E.

[28] Otadi M, Mosleh M (2017). Universal approximation method for the solution of integral equations. Math. Sci..

[29] LeCun Y, Bottou L, Bengio Y, Haffner P (1998). Gradient-based learning applied to document recognition. Proc. IEEE..

[30] Reed, S., de Freitas, N. Neural programmer-interpreters. Available at: https://arxiv.org/abs/1511.06279 (2015).

[31] Luo C, Zhan J, Wang L, Yang Q (2018). Cosine normalization: Using cosine similarity instead of dot product in neural networks. Proc. Int. Conf. Artif. Neural Netw..

[32] Zhang X, Park JC, Salant J (2008). A multiplicative model for spatial interaction in the human visual cortex. J. Vis..

[33] Swindale NV (2008). Feedback decoding of spatially structured population activity in cortical maps. Neural Comput..

[34] Naci L, Taylor KI, Cusack R (2012). Are the senses enough for sense? Early high-level feedback shapes our comprehension of multisensory objects. Front. Integr. Neurosci..

[35] Chollet, F. Keras: Deep learning library for theano and tensorflow. Available at: https://github.com/fchollet/keras (2015)

[36] Basha S, Dubey SR, Pulabaigari V, Mukherjee S (2020). Impact of fully connected layers on performance of convolutional neural networks for image classification. Neurocomputing..

